# Health-related quality of life of salvage prostate reirradiation using stereotactic ablative radiotherapy with urethral-sparing

**DOI:** 10.3389/fonc.2022.984917

**Published:** 2022-10-06

**Authors:** Carlo Greco, Oriol Pares, Nuno Pimentel, Vasco Louro, Beatriz Nunes, Justyna Kociolek, Joao Marques, Zvi Fuks

**Affiliations:** ^1^ The Champalimaud Centre for the Unknown, Department of Radiation Oncology, Lisbon, Portugal; ^2^ Memorial Sloan Kettering Cancer Department of Radiation Oncology Center, New York, NY, United States

**Keywords:** reirradiation, prostate cancer, salvage ablative therapy, SABR, quality of life

## Abstract

**Purpose:**

To explore whether prostate motion mitigation using the rectal distension-mediated technique is safe and effective in stereotactic ablative radiation therapy (SABR) salvage treatment of intraprostatic cancer recurrences following initial radiotherapy for primary prostate cancer.

**Materials and methods:**

Between July 2013 and December 2020, 30 patients received salvage SABR for ^68^Ga- PSMA-11 PET/CT-detected intra-prostatic relapses. Median time from primary RT to salvage reirradiation was 70.2 (IQR, 51.3-116.0) months. Median PSA at retreatment was 3.6 ng/mL (IQR, 1.9-6.2). Rectal distension-mediated SABR was achieved with a 150-cm^3^ air-inflated endorectal balloon and a Foley catheter loaded with 3 beacon transponders was used for urethra visualization and on-line tracking. MRI-based planning employed a 2-mm expansion around the planned target volume (PTV), reduced to 0-mm at the interface with critical organs at risk (OARs). Volumetric Modulated Arc Therapy (VMAT) permitted a 20% dose reduction of the urethra. VMAT simultaneous integrated boost (SIB) of the dominant intraprostatic lesion was deployed when indicated. Median SABR dose was 35 Gy (7 Gy per fraction over 5 consecutive days; range 35-40 Gy). Toxicity assessment used CTCAE v.4 criteria.

**Results:**

Median follow-up was 44 months (IQR, 18-60). The actuarial 3- and 4-year biochemical relapse free survival was 53.4% and 47.5%, respectively. Intraprostatic post-salvage relapse by PSMA PET/CT was 53.3%. Acute grade 2 and 3 genitourinary (GU) toxicities were 20% and 0%, respectively. There were no instances of acute grade ≥2 rectal (GI) toxicity. Late grade 2 and 3 GU toxicities occurred in 13.3% and 0% of patients, respectively. There were no instances of grade ≥2 late rectal toxicity. Patient-reported QOL measures showed an acute transient deterioration in the urinary domain 1 month after treatment but returned to baseline values at 3 months. The median IPSS scores rose over baseline (≥5 points in 53% of patients) between month 6 and 12 post-treatment as a result of urinary symptoms flare, eventually receding at 18 months. The bowel domain metrics had no appreciable changes over time.

**Conclusion:**

Pursuit of local control in intraprostatic failures is feasible and can be achieved with an acceptably low toxicity profile associated with effective OAR sparing.

## Introduction

Dose-escalated fractionated radiotherapy in primary organ confined prostate cancer increases the rates of local tumor control in a dose-dependent manner, also resulting in an associated mitigation of metastatic dissemination (1–3). Nonetheless, PSA failures have been reported in >20% of patients treated with ablative schemes (4). The temporal associations between PSA failure, local relapse, and metastatic spread, however, remain only partially defined. Biopsy studies reported 20–40% intraprostatic cancer-positive findings at 2–3 years after completion of radiotherapy, frequently without concomitant evidence of biochemical or imaging recurrence (4–8), while metastatic spread was reported within a median of 5.4 years after PSA relapse date (4, 9). In fact, 67% of positive biopsies were recorded prior to PSA relapse (Phoenix definition, nadir PSA+2 ng/ml) and positivity risk was independent of biochemical failure in a multivariate regression model (8). Whereas discrimination between a late responding *versus* a relapsing tumor in biopsy positive cases is frequently infeasible, rescue of a potentially recurring lesion is generally not pursued prior to overt PSA relapse. Hence, whether a timely deployed re-irradiation to rescue locally failing cancer might mitigate the later development of metastatic spread remains unanswered. Notably, most available data were generated prior to the prostate-specific membrane antigen (^68^Ga-PSMA) PET scanning era. At present, PSMA might be used for early detection of a relapsing tumor and in differentiating between an intra-prostatic *versus* metastatic failures, providing an opportunity for early rescue of local relapses by prostate re-irradiation (10).

The discovery that the radiobiological linear quadratic (LQ) phenotype of human prostate cancer is characterized by a low (≤2 Gy) *α*/*β* ratio (11) has revolutionized the curative approach to prostate cancer radiotherapy. Prostate cancer *α*/*β* is lower than the functional *α*/*β* ratio of the rectal and urinary mucosae (12, 13), rendering an unprecedented therapeutic ratio, provided extreme hypofractionation is employed (14). Consistent with this notion, recent phase **II** clinical trials have reported ≥95% 5-7 year PSA relapse-free survival (bRFS) with low toxicity rates in prostate cancer patients with NCCN low-risk and favorable intermediated-risk (FIR) disease treated with 5-fraction regimens of 35-40 Gy stereotactic ablative radiotherapy (SABR) (15–17). However, in unfavorable intermediate-risk (UIR) and high-risk patients (e.g. ISUP groups ≥3) similar SABR schedules rendered only approximately 65-75% 5-7 year bRFS (15–18). Furthermore, ^68^Ga-PSMA studies have indicated that relapses occur primarily within the prostate, with or without extra-prostatic spread (19), indicating tumor progression during clonal expansion may be associated with evolution of radioresistant phenotypes. This notion is consistent with emerging data suggesting an existence of *α*/*β* heterogeneity in prostate cancer (20), ranging between 1.3 and 11.1 Gy (21). Per a given hypofractionation scheme, prostate tumor clonogens operating a high *α*/*β* ratio would be exposed to a significantly reduced BED as compared to those operating an *α*/*β* ratio of ≤2 Gy. Hence, the optimal management of UIR and high-risk prostate cancer with extreme hypofractionated radiotherapy needs to be reevaluated. Regardless of the feasibility and outcome of alternative therapies, at present, there is an increasing interest for salvage re-irradiation of PSMA-detected local relapses even before PSA relapse becomes apparent, especially since early predictors of pending relapse after prostate cancer radiotherapy are emerging (22).

Salvage radical prostatectomy for radiation-recurrent prostate cancer is no longer considered the option of choice, because of long post-surgical functional recovery, in particular, in advanced age, high frequency of positive margins, and up to 25% serious complications, including anastomotic strictures (23). Salvage brachytherapy has so far been the most explored re-irradiation modality, despite the limitations associated with an invasive technique, and the restrictions obliged by age and by comorbidities (24, 25). However, more recent experience indicates that high dose-rate brachytherapy (HDR-BT) and re-irradiation with low biological effective dose (BED) extreme hypofractionation (24-36 Gy in 5-6 fractions) render similar bRFS rates and tolerable late toxicities (24, 25). In particular, salvage SBRT is attracting increasing attention despite the restricted dose and concerns of re-irradiating previously exposed normal organs at risk (24–27). Nonetheless, a pursuit of non-toxic control of relapsing disease with high-BED hypofractionation remains a therapeutic target for radioresistant high-risk prostate cancer phenotypes.

The present study addresses this issue. We have recently reported that high- BED in extreme hypofractionated radiotherapy (5 x 9Gy SABR) affords low toxicity outcomes in locally confined prostate cancer when the rectal distention-mediated technique is used (18, 28, 29). The rectal distention-mediated protocol affords intra-fractional prostate motion mitigation, urethral sparing *via* inverse dose painting, and high precision in daily targeting of the PTV, conformally avoiding the rectum (29). The specific aim of the present study was to evaluate the toxicity of rectal distention mediated SABR when employed in rescue re-irradiation of intraprostatic lesions relapsing after different initial radiation protocols.

## Materials and methods

### Patients

Eligible biochemically recurrent patients were treated with salvage SABR to the prostate following confirmation of intraprostatic recurrence by imaging after primary external-beam (EBRT) and/or brachytherapy with a minimum interval of 24 months since primary therapy. Biochemical recurrence after primary RT was defined according to the Phoenix definition as a rise >2 ng/mL above nadir. Patients with a medical history of prostate surgery were not included. Patients with persistent rectal or urinary symptoms grade ≥2 were excluded.

### PET/CT staging and identification of intraprostatic index lesion

Patients with a biochemical relapse were assessed with a ^68^Ga-labelled prostate-specific membrane antigen (^68^Ga-PSMA)-11 PET/CT scan to determine whether the relapse was solely intraprostatic *vs* intra- *and* extra- *vs* exclusively extra-prostatic with nodal and/or distant dissemination. An activity of 2 MBq/kg of patient body weight of ^68^Ga-PSMA-11 was administered using an automatic injector (INTEGO™, MEDRAD) and images were acquired at approximately 60 minutes post-injection. The PET/CT (Gemini TF, Philips) scan was acquired with a low-dose CT (120-140 kV, 60 mA per rotation) from the skull base to the upper third of the thighs. PET data were obtained thereafter with a sequence of 6 to 8 bed positions, always on 3D mode for 1.5 to 3 minutes on average per bed position. In addition to visual analysis, quantitative standardized uptake value (SUV) evaluation was performed within the volumetric region of interest (Extended Brilliance Workspace algorithm NM 2.0 AB - V5.4.3.40140, Philips). The SUV for the voxel with the highest activity concentration (SUV_max_) was recorded. Institutional criteria for quantitative assessment ^68^Ga-PSMA uptake were: SUV_max_ of lesion/SUV_max_ of normal prostate or surrounding tissues >4.0 was considered positive, 2.0 to 4.0 suspicious and < 2.0 negative.

In addition to ^68^Ga-PSMA-11 PET/CT, multiparametric MRI scans of the prostate and biopsy were employed where appropriate. Histologic confirmation of local recurrence was requested in all cases but not always obtained due to patient refusal. The area of pathologic ^68^Ga-PSMA avidity was used to identify a boost volume for dose escalation whenever feasible.

### Treatment planning and delivery

Patient set-up, treatment planning and treatment delivery were performed according to our established protocols developed for primary prostate SABR (29, 30). All patients received a microenema to empty their bowel and voided their bladder before planning procedures and treatment. Patients were planned in the supine position with leg fixation. Catheterization with a 12 French gauge (4 mm diameter) Foley catheter with 3 embedded beacon transponders (Calypso, Varian Medical Systems, Palo Alto, CA) was performed for intra-fractional target tracking. The Foley catheter was also used to guide segmentation of the whole length of the prostatic urethra for optimal sparing. Prostate immobilization was achieved by insertion of an endo-rectal balloon (Rectal Pro, QLRAD Inc., FL) inflated with 150 cm^3^ of air (29, 30). A CT and a T2W 3D MRI scan were acquired in treatment position.

The planning CT and MRI scans were fused to delineate the prostate CTV and OARs. The PET/CT was fused to the planning image set to identify a tracer-avid boost volume wherever deemed useful at the discretion of the treating physician. The PTV consisted of the CTV with an anisotropic 2 mm expansion margin, reduced to 0 mm at interface with the rectal wall, the bladder, the urethra wall (defined as a 2 mm expansion around the catheter), the urogenital diaphragm (UGD) and the neurovascular bundles (NVB). Inverse dose-painting permitted effective OAR sparing, predicated upon the reproducible high-precision positioning of the target and all OARs at every treatment session on account of the organ motion mitigation protocol. The urethral wall was negatively dose-painted to fulfill D_2%_ ≤31.5 Gy and D_1cm_^3^ ≤28 Gy. Dose constraints for the rectal wall were D_2%_ ≤35 Gy, D_50%_ ≤21 Gy and D_1cm_^3^ ≤31.5 Gy. Dose constraints for the bladder were D_2%_ ≤35 Gy, D_50%_ ≤17.5Gy and D_1cm_^3^ ≤35 Gy. The dose to the UGD was constrained at D_2%_ ≤31.5 Gy. Due to the high inherent dose heterogeneity of the plan dose prescriptions, PTV doses are reported in accordance with the International Commission on Radiation Units (ICRU) recommendations (31) as D_50%._ Priority was given to OAR sparing, but for the PTV a D_50%_ ≥35 Gy was pursued. A 40 Gy simultaneous integrated boost (SIB) to the PET/CT-identified dominant intraprostatic lesion (DIL) and topography of positive biopsies, when available, was pursued wherever feasible.

Plans were optimized for PTV dose coverage and OARs constraints with the progressive resolution optimizer (PRO v10.0.28 - v13.7.14 in Eclipse, Varian Medical Systems, Palo Alto, CA), calculated with the analytical anisotropic algorithm (AAA v10.0.28 - v13.7.14). A 10 MV flattening filter-free (FFF) beam energy and 4 volumetric modulated arc therapy (VMAT) arcs were used in all cases.

All treatments were delivered in 5 fractions on a linear accelerator with a 2.5 mm leaf width multi leaf collimator (TrueBeam STx or EDGE, Varian Medical Systems, Palo Alto, CA). All treatment plans were quality assured using an ArcCHECK phantom (Sun Nuclear Corp. FL) to fulfill a gamma (3%/3mm) passing rate >90% objective according to AAPM guidelines.

**Figure 1:**
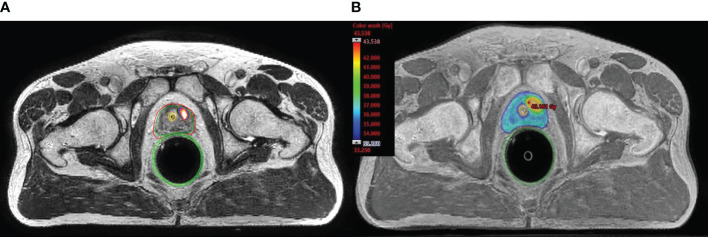
Typical planning fused image set (MRI and PSMA-PET/CT) and dosimetric results. **(A)** shows MRI/PET-CT fusion with identification of intraprostatic tracer-avid recurrence. **(B)** shows color-wash dose distributions representative of the typical plan with the boost dose (40 Gy) covering the dominant lesion.

On-board cone-beam computed tomography (CBCT) was used prior to treatment delivery to assure reproducible patient set-up and target localization. Target position discrepancies of ≥1 mm in translation or ≥1 degree in rotation were corrected *via* a 6-degrees of freedom couch (PerfectPitch 6-DoF Couch, Varian Medical Systems, Palo Alto, CA). If beacon-transponder signals exceeded an accepted 2 mm deviation threshold for ≥5 seconds, treatment was interrupted, and treatment target position was adjusted by repeat CBCT. Patients received treatment daily over 5 consecutive days or every other day over 10 days according to treating physician’s preference. **Figure 1A** shows MRI/PET-CT fusion with identification of intraprostatic tracer-avid recurrence and **Figure 1B** shows dose distributions representative of the typical plan with the boost dose covering the dominant lesion.

### Toxicity and quality of life assessment

Toxicity (NCI CTCAE v.4.0) was assessed post-treatment at one month and every 3 months to 12 months (+/- 4 weeks), at every 6 months thereafter. Acute toxicity was defined as any adverse event occurring within 3 months from the beginning of treatment. Patient-reported IPSS and EPIC-26 questionnaires were completed at baseline and at the above time points.

### Statistical methods

The primary endpoints of the present study were incidence of treatment-related acute and late adverse events and PSA outcomes. The second biochemical relapse-free survival was defined according to the Phoenix criteria (nadir + 2 ng/mL). Median follow-up was calculated from salvage SABR until biochemical recurrence or demise. Actuarial bRFS, metastasis-free survival (MFS), GU and GI toxicities, and patient-reported quality of life (QOL) scores were computed from the end of treatment using the Kaplan-Meier method. For each EPIC domain, a summary score was calculated at each of the study time-points. The association between toxicity and risk factors was analyzed by the exact Fisher test for categorical variables and by the Wilcoxon Mann Whitney test for continuous variables. The clinically meaningful decline in QOL (minimally important difference, MID), was defined as one-half of the standard deviation from baseline for each domain. Univariate analysis of relevant variables was performed using the Cox proportional hazards regression method. Hazard ratio (HR), 95% confidence intervals (CI) were obtained, and the level of statistical significance was set at alpha =0.05. Statistical computations were performed using the GraphPad *Prism 7.0* software (Prism Inc, Reston, VA).

## Results

The present analysis includes 30 eligible patients who received salvage SABR for failing intra-prostatic lesions between July 2013 and December 2020. All but two patients were referred from outside institutions after biochemical failure. Patient and tumor characteristics at the time of primary irradiation are summarized in **Table 1**. Median age was 66.3 years (IQR, 61.5-70.9). Primary RT to the prostate was given solely with low dose rate (LDR) brachytherapy in 11 patients (36.6%), with combined pelvic EBRT and LDR in 2 (6.7%) or EBRT exclusively in 17 patients (56.7%).

**Table 1:** Patient and tumor characteristics at initial treatment.

Characteristics	(n=30)
Age, y	
median (IQR)	66.3 (61.5-70.9)
iPSA, ng/mL	
median (IQR)	8.7 (6.8-10.4)
ISUP Grade, n (%)	
Group 1 (3+3)	11 (36.7)
Group 2 (3+4)	12 (40.0)
Group 3 (4+3)	4 (13.3)
Group 4 (4+4)	1 (3.3)
Unkown	2 (6.7)
T-stage, n (%)	
T1c	4 (13.3)
T2a	4 (13.3)
T2b	10 (33.4)
T2c	9 (30.0)
T3b	1 (3.3)
Unknown	2 (6.7)
NCCN Risk, n (%)	
Low	2 (6.7)
Favorable intermediate	11(36.6)
Unfavorable intermediate	12 (40.0)
High	3 (10.0)
Unknown	2 (6.7)
Initial Radiotherapy	
LDR brachytherapy	11 (36.6)
EBRT + LDR brachytherapy	2 (6.7)
EBRT	17 (56.7)
EBRT dose	
(Gy) median (IQR)	74 (71.6-74)
ADT n (%)	16 (57.9)
duration (mo) median (IQR)	18 (6-27)

PSA, Prostate Specific Antigen; iPSA, initial PSA; ADT, androgen deprivation therapy; IQR, interquartile range; mo, months; y, years.

Patient and salvage SABR characteristics are summarized in **Table 2**. Median time from primary RT to biochemical failure and to salvage reirradiation were 55.8 (IQR, 46.0-83.5) and 70.2 (IQR, 51.3-116.0) months, respectively. Median age at the time of salvage treatment was 71.7 (IQR, 76.6-78.6) years. Median PSA at the time of retreatment was 3.6 ng/mL (IQR, 1.9-6.2). All included patients had a ^68^Ga-PSMA-11 PET/CT-documented intraprostatic relapse; 76.7% (n=23) exhibited intraprostatic relapses exclusively and 23.3% also had extraprostatic regional nodal (n=4) or distant (n=3) oligoprogression, deemed amenable to ablative irradiation. At the discretion of the referring physician, 53.3% (n=16) patients received androgen deprivation therapy (ADT) at the time of biochemical failure for a median duration of 15 months (IQR, 6-34). A total of 19 patients (63.3%) had a confirmatory positive prostate biopsy at the time of recurrence. In 6 cases pathology grade was not reported due to marked radiation changes, but the pathology report unambiguously confirmed presence of viable adenocarcinoma. All but 1 case where grade could be established (12/13; 92.3%) exhibited a shift towards a higher biopsy ISUP grade (*e.g.* from ISUP grade 1-2 to ≥3, or grade 3 to ≥4).

**Table 2:** Patient and tumor characteristics at salvage reirradiation treatment.

Characteristics	(n=30)
Age, (y)	
median (IQR)	71.7 (76.6-78.6)
Nadir PSA after initial RT	
(ng/mL) median (IQR)	0.68 (0.2-1.3)
Time to recurrence	
(mo) median (IQR)	55.8 (53.0-83.5)
PSA at recurrence	
(ng/mL) median (IQR)	3.8 (2.9-4.8)
Time from primary irradi	
(mo) median (IQR)	70.2 (51.3-116.0)
iPSA before salvage SABR	
(ng/mL) median (IQR)	3.6 (1.9-6.2)
ISUP Grade, n (%)	
Group 2 (3+4)	2 (6.7)
Group 3 (4+3)	3 (10.0)
Group 4 (4+4)	4 (13.3)
Group 5 (4+5, 5+4, 5+5)	4 (13.3)
Grade not reported	17 (56.7)
Prostate volume at retreatment	
(cm^3^) median (IQR)	26.7 (24.0-39.3)
Site of recurrence on PET/CT n (%)	
Intraprostatic only	23 (76.7)
Intraprostatic + pelvic nodes	4 (13.3)
Intraprostatic + oligomets	3 (10.0)
SABR 5 fractions n (%)	30 (100)
Dose/fr (Gy) n (%)	
Whole gland 7 Gy	18 (60.0)
Whole gland 7 Gy + SIB 8 Gy	8 (26.6)
Whole gland 8 Gy	2 (6.7)
Partial prostate 8 Gy	2 (6.7)
ADT n (%)	16 (36.4)
duration (mo) median (IQR)	15 (6-34)

PSA, Prostate Specific Antigen; iPSA, initial PSA; ADT, androgen deprivation therapy; IQR, interquartile range; mo, months; y, years; BED, biological equivalent dose; SIB, simultaneous integrated boost.

All patients were strictly planned and treated with the distention-mediated prostate immobilization technique and a beacon transponder-loaded Foley catheter was introduced to monitor intrafraction target motion. Plan objectives and dosimetric results are summarized in **Table 3**. Considering an α/β ratio of 2 Gy, the BEDs for the 35 Gy and 40 Gy (whole gland or SIB) plans translate into 157 Gy and 200 Gy, respectively, corresponding to an equivalent dose at 2 Gy (EQD_2_) of 78.75 Gy and 100 Gy, respectively.

**Table 3:** Plan dosimetry.

			
structure	plan objective	median	mean	IQR
PTV				
D_50%_ (Gy)	≥ 35.0	36.4	36.9	36.6-36.3
D_mean_ (Gy)	≥ 35.0	36.2	36.7	36.7-36.0
D_95%_ (Gy)	≥ 31.5	33.5	33.6	33.9-32.7
D_2%_ (Gy)	≤ 37.5	37.5	39.3	41.6-37.3
D_98%_ (Gy)	≥ 29.7	31.8	31.8	32.4-31.1
V_35Gy_ (%)	≥ 80	89.	83.7	91.0-85.0
V_31.5Gy_ (%)	≥ 95	97.5	98.3	98.9-97.1
Urethral wall				
D_2%_ (Gy)	≤ 31.5	30.2	29.6	35.0-29.9
D1cm^3^ (Gy)	≤ 28.0	26.2	24.3	26.5-24.8
Bladder				
D_2%_ (Gy)	≤ 31.5	32.1	28.0	32.6-28.8
D_50%_ (Gy)	≤ 17.5	4.6	6.5	10.5-2.0
D1cm^3^ (Gy)	≤ 31.5	33.1	30.7	33.9-32.1
Rectal wall				
D_2%_ (Gy)	≤ 37.5	30.9	30.1	31.4-30.6
D_5%_ (Gy)	≤ 31.5	29.3	27.4	29.8-27.6
D_50%_ (Gy)	≤ 17.5	7.9	6.9	9.4-4.9
D_1cm3_ (Gy)	≤ 28.0	30.7	29.8	31.0-30.0
UGD				
D_2%_ (Gy)	≤ 33.3	30.2	27.7	31.8-26.7
Penile bulb				
D_2%_ (Gy)	< 28.0	2.6	3.8	5.1-1.4
D1cm (Gy)	≤ 17.5	1.2	1.7	2.3-0.8
NVBs				
D_2%_ (Gy)	< 35.0	36.6	36.1	37.0-36.6
D_50%_ (Gy)	≤ 24.5	25.6	26.2	29.9-24.8
Femoral heads				
D_2%_ (Gy)	≤ 17.5	6.9	6.8	8.3-5.0

PTV, Planning Target Volume; D_mean_, mean dose; D_2%_, D_5%,_ D_50%_D_95%,_ D_98%,_ minimum dose to n% of the structure; V_35Gy_, V_31.5Gy_, percentage of structure receiving 35Gy or 31.5Gy (100% and 90% of the prescription dose); D1cm^3^, dose to 1 cm^3^ of the structure; UGD, urogenital diaphragm; NVB, neurovascular bundles.

Whole-gland salvage SABR dose was 35 Gy in 5 fractions in 26 patients (86.7%). In 2 patients (6.7%) the whole-gland prescription dose was 40 Gy. In 8 of the 35 Gy whole-gland patients, dose was escalated to 40 Gy with SIB to the dominant tracer-avid lesion. Two patients (6.7%) received partial gland (≥50% of the prostate) treatment to the region of tracer-avidity. In summary, dose prescription to PSMA-detected intraprostatic lesions had BED_2_ <160 Gy *vs >*160 Gy in 60.0% and 40.0% of patients, respectively.

The first 4 patients in this series (13.3%) received treatment every other day over 10 days and the subsequent 26 (86.7%) patients were treated over 5 consecutive days (*i.e.* Monday through Friday). There was no difference in toxicities between the two regimens. In addition to local treatment, patients with extraprostatic disease sites were treated with an ablative attempt to all sites of tracer-avid disease with single dose radiotherapy (SDRT) with a prescription dose of 24 Gy as per institutional guidelines (32). Median follow-up time was 44 months (IQR, 18-60).

### Adverse events

Acute urinary (GU) grade 1-2 symptoms peaked at 1-month post-treatment with an overall incidence of 60% (18/30) largely consisting of dysuria and frequency. Acute grade 2 GU toxicity was observed in 20% (6/30) patients, including 2 cases (6.7%) of retention requiring catheterization during the first month post-therapy. There were no cases of acute grade 3 GU toxicity. Acute grade 1 rectal (GI) toxicity occurred in 20% (6/30) of cases and was mostly represented by tenesmus. There were no instances of acute grade ≥2 GI adverse events.

Late grade 1 or 2 GU toxicities occurred, in 36.7% (11/30) and 13.3% (4/30) patients, respectively, at a median of 7.2 months (IQR, 6.1-8.0). There were no instances of grade 3 toxicity. None of the patients developed late urinary retention requiring catheterization. The actuarial cumulative incidence of grade 2 GU toxicity was 16.4%. The occurrence of grade 2 urinary toxicity was not associated with the use of a fraction size of 8 Gy (*i.e.* BED_2_ >160 Gy whole gland or SIB) (*p* = 0.64; HR 0.62, 95% CI 0.08-4.79). On univariate analysis, the only variable associated with occurrence of grade 2 urinary toxicity was gland volume at reirradiation. Patients with gland volumes above the median (26 cm^3^) had a 32.3% actuarial probability of developing grade 2 urinary symptoms *vs* 0% for those with smaller volumes (*p* = 0.004; HR 0.13; 95% CI 0.01-0.94). Late grade 1 rectal toxicity occurred in 20% (6/30) of patients at a median of 9.1 months (IQR, 7.0-13.0) post-therapy, largely consisting of tenesmus with only one patient experiencing sporadic rectal bleeding. There were no instances of grade ≥2 rectal toxicity.

### Patients reported quality of life

**Figure 2:**
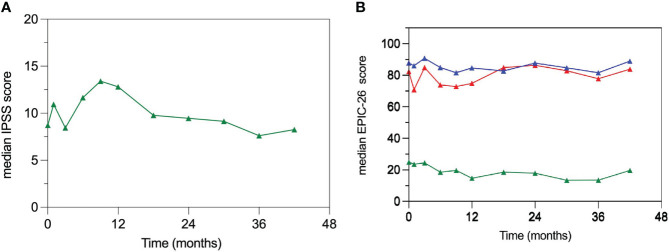
Patient-reported quality of life (QOL) measures. **(A)** Median IPSS scores; there is a significantly increased median IPSS score in the interval between 6 and 12 months post-treatment returning to baseline values at month 18. **(B)** Median EPIC-26 summary scores for the urinary (red), bowel (blue) and sexual (green) domains. EPIC 26 summary scores are comprised between 0 and 100 with higher scores indicating better QOL.

The median baseline IPSS score was 7.5 (IQR, 5.8-13) and 43.5% of patients had moderate to severe (IPSS ≥ 8) lower urinary tract symptoms (LUTS) before reirradiation. Patient-reported QOL measures showed an acute transient deterioration in the urinary domain after treatment (**Figures 2A, B**). The 1-month median IPSS score increased to 11.5 points (IQR, 8.3-14.3) but the difference did not reach statistical significance (*p* = 0.20). However, the 1-month median GU EPIC-26 summary score drop was 8.5 points (IQR, -24 - 0) with a statistically significant difference from baseline (*p* = 0.018). Both, metrics returned to baseline values at 3 months. However, in the interval between month 6 and 12 post-treatment the median IPSS scores rose over baseline (≥5 points in 53% of patients) as a result of urinary symptoms flare, eventually receding at 18 months (**Figure 2A**). Consequently, the GU EPIC-26 summary scores declined, reflecting urinary domain deterioration, in the interval between month 6 and 12 (**Figure 2B**). Both QOL metrics returned to baseline values at month 18 and appeared to remain relatively stable thereafter. The bowel domain EPIC-26 summary score had no appreciable changes over time.

### PSA outcomes

Median nadir PSA (nPSA) in the entire cohort was 0.33 ng/mL (IQR, 0.12-0.89) at median time of 8.3 months (IQR, 4.7-13) from the end of salvage SABR. Median nPSA was 0.89 ng/mL (IQR, 0.3-1.7) for ADT-naïve patients *vs* 0.30 ng/mL (IQR, 0.23-0.46) for those who received adjuvant ADT (*p* = 0.02). None of the patients exhibited a PSA bounce of >0.2 ng/mL over nadir. A total of 15 patients (50%) experienced a second biochemical relapse, at a median of 22.4 months (IQR 10.7-46.2) after salvage SABR.

**Figure 3:**
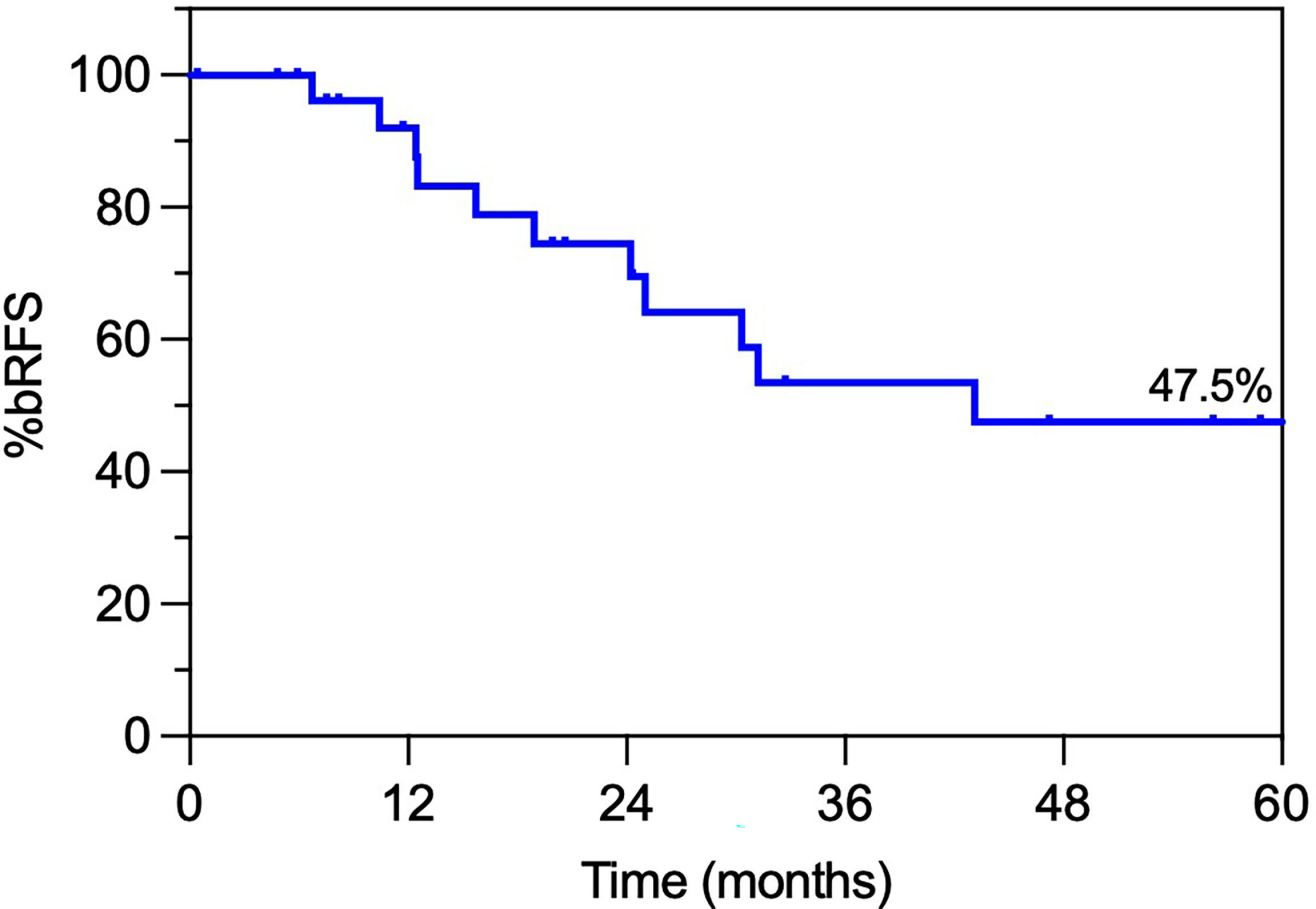
Actuarial prostate specific antigen (PSA) relapse-free survival.

The actuarial bRFS probabilities from the salvage treatment for the entire cohort were 74.5%, 53.4% and 47.5%, at 24, 36 and 48 months, respectively (**Figure 3**). The respective bRFS probabilities for the 23 patients with intraprostatic relapse exclusively were 72.6%, 52.8% and 45.3%. Importantly, in the subgroup with exclusive intraprostatic recurrence at the time of reirradiation there was a trend towards improved biochemical response in patients treated with BED_2_ >160 (*i.e.* 8 Gy/fr); the 3-year bRFS probabilities were 72.9% *vs* 36.4% for the higher *vs* the lower BED_2_ doses, respectively (*p* = 0.08; HR 0.34, 95% CI 0.10-1.14).

Whereas the number of patients in each sub-cohort was small, there was no statistically significant difference in 36-month bRFS probabilities with the use of ADT *vs* no ADT at reirradiation (64.3% *vs* 40.0%; *p* = 0.58; HR 0.71; 95 CI 0.21-2.38). Interval since initial treatment and reirradiation was not significantly associated with bRFS (*p* = 0.85; HR 0.90; 95 CI 0.31-2.59). NCCN risk group at primary RT (low-risk and FIR *vs* UIR and high-risk) did not appear to be associated with bRFS (*p* = 0.19; HR 1.93, 95 CI 0.69-5.35). However, primary irradiation modality (BT *vs* EBRT) was associated with statistically different bRFS (3-year bRFS 78.7% vs 31.2% for BT vs EBRT, respectively; *p* = 0.02; HR 0.24; 95% CI 0.07-0.80) possibly reflecting a selection bias for lower grade and stage disease at primary treatment.

### ^68^Ga-PSMA PET/CT characterization of second PSA relapses

All 15 patients with biochemical failure had a staging ^68^Ga-PSMA PET/CT at the time of relapse. Of these, 4 (26.7%) presented an intraprostatic relapse only at the same site of the initially relapsing dominant lesion, 4 patients (26.7%) had both extra-prostatic progression and intraprostatic relapse, 3 of whom relapsing at the site of the salvage DIL, and 7 patients (46.7%) had extra-prostatic progression only. Thus, the overall rate of intraprostatic relapse at the site of salvage DIL was 23.3% (7/30). Patterns of recurrence based on ISUP grade was not feasible because paucity of information due to the presence of severe radiation-induced changes.

## Discussion

The present study provides compelling evidence that employing the rectal distension-mediated technique in prostate cancer SABR promotes OAR preservation even in the salvage reirradiation setting. The use of rectal distension and urethral-sparing limits the acute and late GU and GI toxicities to grade ≤2, despite escalation of the prostate reirradiation dose to 35-40 Gy in 5 daily fractions. Patient-reported health-related measures confirm the long-term preservation of quality of life with our salvage reirradiation protocol. Although this study represents a small single-institution experience, the clinical outcomes compare favorably with published reirradiation series of 25-35 Gy SABR (26, 27, 33–36). A recent multi-institutional series of 100 patients treated with a salvage 6-fraction median dose of 36 Gy reported, a 3-year second bRFS of 55% and an actuarial 20.8% 3-year grade ≥2 GU and GI toxicities (27). Importantly, multivariate analysis showed that the dose prescription was a predictor of bRFS, as patients receiving reirradiation with BED >120 Gy fared better than lower BED doses, in line with the present series showing a trend towards improved bRFS with higher doses. Another prospective salvage reirradiation trial of 50 patients with biopsy-proven recurrent prostate cancer retreated with 34 Gy in 5 fractions to the whole prostate (26), reported a 60% 5-year bRFS, albeit with GU late grade ≥3 toxicity of 8%, underscoring the need for more effective sparing of OARs. Hence, although our technique of high-dose 5-fraction salvage SABR of the whole prostate did not yield higher biochemical control than those reported by others, we demonstrate lower toxicity and quality of life preservation by employing the rectal distension-mediated protocol in salvage prostate reirradiation.

The use of ^68^Ga-PSMA PET/CT in the present study indicates that approximately 25% of initially relapsing lesions still recur post-reirradiation at their original relapse sites. Emerging evidence indicates that unfavorable intermediate risk (UIR) and high-risk prostate tumors (IUSP grade ≥3) exhibit approximately 20% cumulative incidence of PSMA-detected intraprostatic local relapse following 45 Gy 5-fraction primary SABR (18). This observation hypothetically suggests that an intraprostatic recurrent lesion might frequently represent high *α*/*β* radioresistant genetic mutant clone, while the native low *α*/*β* radiosensitive foci might be permanently ablated by ultra-high dose SABR (18, 21). While no existing pathological or metabolic biomarkers can predict upfront the existence of radioresistant clones in an individual patient, the recent introduction of ^68^Ga-PSMA in directing DIL dose painting may partially compensate for this uncertainty (37–41). The safety and effectiveness of SIB/DIL in the context of SABR reirradiation will need to be tested in carefully designed clinical trials.

Due to the risk of toxicity of whole prostate reirradiation, some investigators have adopted partial prostate reirradiation, predicated upon the limited multifocal nature of recurrent prostate cancer and the overwhelming predominance of clinically significant relapses at the site of the original DIL (42, 43). Molecular PET image guidance has been recently employed in salvage reirradiation with the intent of maximally sparing the previously irradiated prostate and OAR (44). An early analysis of a prospective PSMA-directed focal reirradiation for locally recurrent prostate cancer using 36 Gy in 6 fractions without the use of ADT has recently reported an estimated 80% bRFS rate at 2 years and a 4% late grade 2 GU toxicity (45).

An IRB-approved phase II study is currently underway in our Institution (ClinicalTrials.gov NCT02570919), where ^68^Ga-PSMA-detected lesions are receiving SIB/DIL dose painting to 30 Gy, provided that the OAR dose-constraints are fulfilled for 24 Gy whole prostate SDRT (30). The preliminary outcome data indicate that this treatment is safe and yields encouraging initial responses. Whether focal ^68^Ga-PSMA-guided SDRT could be employed in salvage prostate lesion reirradiation could be tested in prospective studies.

## Conclusion

The approach to salvage reirradiation of intraprostatic relapse is rapidly evolving due to the availability of molecular imaging. Pursuit of local control in selected locally failing prostate cancer is feasible and can be achieved with an acceptably low toxicity profile with effective AOR sparing. The role of focal reirradiation should be tested in prospective clinical trials.

## Data availability statement

The datasets presented in this article are not readily available. Requests to access the datasets should be directed to carlo.greco@fundacaochampalimaud.pt.

## Ethics statement

The studies involving human participants were reviewed and approved by Champalimaud Ethics committee. The patients/participants provided their written informed consent to participate in this study.

## Author contributions

CG developed the concept of the study, participated in data collection, data analysis, article drafting, table/figure creation, and article revision. OP, NP, VL, BN, and JK participated in clinical data collection and data analysis. JM participated in data collection and analysis. ZF is a senior author who participated in data analysis, article drafting, review, and revision. All authors contributed to the article and approved the submitted version.

## Conflict of interest

The authors declare that the research was conducted in the absence of any commercial or financial relationships that could be construed as a potential conflict of interest.

## Publisher’s note

All claims expressed in this article are solely those of the authors and do not necessarily represent those of their affiliated organizations, or those of the publisher, the editors and the reviewers. Any product that may be evaluated in this article, or claim that may be made by its manufacturer, is not guaranteed or endorsed by the publisher.
